# Atmospheric Factors Affecting a Decrease in the Night-Time Concentrations of Tropospheric Ozone in a Low-Polluted Urban Area

**DOI:** 10.1007/s11270-018-4012-x

**Published:** 2018-10-25

**Authors:** Kazimierz Warmiński, Agnieszka Bęś

**Affiliations:** Department of Chemistry, Research Group of Environmental Toxicology, University of Warmia and Mazury in Olsztyn, Prawocheńskiego 17, 10-720 Olsztyn, Poland

**Keywords:** Ozone decomposition, Tropospheric ozone, Urban air pollution, Night-time reaction, Nitrogen oxides, Meteorological conditions

## Abstract

Ozone (O_3_) decomposition in the troposphere is a very important process which prevents excessive O_3_ accumulation in the air. It is particularly significant on warm summer days which are marked by a high risk of photochemical smog. We used Spearman’s rank correlation test to determine relationships between the drop in O_3_ concentrations over time (-ΔO_3_), nitrogen oxide (NO), nitrogen dioxide (NO_2_), and total nitrogen oxide (NO_x_) concentrations and meteorological factors (1-h average) in low-polluted urban area in Olsztyn (north-eastern Poland). Nitrogen oxide concentrations were measured continuously by the chemiluminescence method, and O_3_ concentrations were determined by the UV photometric method. The obtained results suggest that the rate of decomposition of tropospheric O_3_ is affected mostly by the presence of NO_x_, high temperature, and air humidity (positive correlation) as well as by wind speed (negative correlation). Maximum correlation coefficient values were reported between –ΔO_3_ and air temperature, –ΔO_3_ and absolute air humidity when NO_x_ concentrations were low (below 1.0 microgram per cubic meter), reaching 0.271 and 0.243, respectively. These results indicate that O_3_ also reacted with air components other than NO and NO_2_. Precipitation at average temperature of < 0 °C did not significantly contribute to a drop in O_3_ concentrations at night-time. In the warm season, precipitation slowed down the rate of O_3_ decomposition, mostly because NO_x_ were scrubbed by rain. An analysis of seasonal and daily –ΔO_3_ fluctuations revealed that –ΔO_3_ values were highest in the summer and shortly after sunset in the diurnal cycle.

## Introduction

High ozone (O_3_) concentrations in the tropospheric layer of the Earth’s atmosphere pose a threat to the health of humans and land animals and have an adverse effect on plants (de Wit et al. 2015; Harmens et al. 2016). Episodes of high concentrations of O_3_, particulate matter (PM10, PM2.5), and nitrogen dioxide (NO_2_) are major current problems related to air pollution. However, it should be stressed that PM10, PM2.5, and NO_2_ are found mostly in the air in urban and industrial regions, whereas O_3_ is present not only in urban but also in suburban and rural areas. The maximum daily 8-h mean concentration of O_3_ should not exceed 120 μg m^−3^ (long-term objective) or 120 μg m^−3^ on more than 25 days per calendar year (target value) (EU 2008). In Europe, 41% of all stations showed O_3_ concentrations above the target value for the protection of human health in 2015, which is considerably more stations than over the previous 5 years. In addition, only 13% of air quality monitoring stations fulfilled the long-term objective (no exceedance of the threshold level). Eighty-eight percent of the stations with values above the long-term objective were background stations (EEA 2017). Tropospheric O_3_ is also a greenhouse gas which contributes to global warming (IPCC 2007). In addition to its adverse effect on human health and the natural environment, tropospheric O_3_ also contributes to significant economic losses by damaging crops, lowering crop yield and causing damage to certain materials, in particular rubber. The annual losses resulting from the above have been estimated at around €6.7 billion in 47 countries in Europe (Holland et al. 2006).

Large quantities of O_3_ are formed in the stratosphere. A part of this naturally formed stratospheric O_3_ goes down to troposphere. However, photochemical processes involving nitrogen oxides (NO_x_), volatile organic compounds (VOCs), and carbon monoxide (CO) are a more important source of O_3_ in ground-level ambient air (Sharma et al. 2017). Photochemical smog is the most dangerous phenomenon related to the formation of O_3_ as a result of the above chemical reactions. In addition to O_3_, a smoggy atmosphere comprises other characteristic components such as organic oxidizers (including peroxyacetyl nitrate, PAN), aldehydes, NO_x_, hydrogen peroxide, and free radicals (Kanaya et al. 2007). Photochemical smog is one of the most adverse contributors to the quality of urban air (EEA 2017). Its formation is supported by the following meteorological conditions: high insolation, high temperature, temperature inversion and dry, stagnant air masses (Manahan 2007; Hosoi et al. 2011). This environment triggers intense reactions with sunlight irradiation which produce O_3_ and other secondary air pollutants. The most important phase of those changes is the NO–NO_2_–O_3_ inter-conversion cycle which takes place in line with the below equations (Crutzen et al. 1999; Warmiński and Bęś 2009):

R1 $$ N{O}_2\overset{h\upsilon}{\to } NO+O $$

R2 $$ O+{O}_2+M\to {O}_3+M $$

R3 $$ NO+{O}_3\to N{O}_2+{O}_2 $$

where

M: molecules which absorb excess energy and determine the stability of O_3_ molecules (usually N_2_ or O_2_ molecules)
*hν*: photon energy (with electromagnetic wave length of λ = 290–430 nm)

R1-R3 reactions establish a photostationary state equilibrium which is described by the following equation (Leighton 1961):

1 $$ \frac{\left[\mathrm{NO}\right]\left[{\mathrm{O}}_3\right]}{\left[{\mathrm{NO}}_2\right]}=\frac{J_{N{O}_2}}{k_3} $$

where

$$ {J}_{N{O}_2} $$
nitrogen dioxide (NO_2_) photolysis rate constant (rate constant of R1 reaction)
*k*_*3*_: nitrogen oxide (NO) oxidation rate constant (rate constant of R3 reaction)

The above equation demonstrates that all other reactions which reduce NO concentrations in favor of NO_2_ increase O_3_ concentrations. Those reactions are inclusive of photochemical oxidation of CO, methane (CH_4_), and non-methane VOC (Warmiński and Rogalski 2007; Kalbarczyk and Kalbarczyk 2017), namely,

R4 $$ CO+2{O}_2\overset{N{O}_x\; h\nu}{\to }C{O}_2+{O}_3 $$

R5 $$ C{H}_4+4{O}_2\overset{N{O}_x\; h\nu}{\to } HCHO+2{O}_3+{H}_2O $$

Atmospheric O_3_ is a relatively unstable gas. It participates in a variety of redox reactions in the atmosphere. Atmospheric O_3_ reacts with both organic and inorganic compounds (mainly with NO, SO_2_, CO, H_2_O) which are in plentiful supply in ground-level air (Crutzen et al. 1999; Hosoi et al. 2011). For this reason, cyclic, diurnal changes are observed in tropospheric O_3_ levels where maximum concentration values are reported in the afternoon and minimum concentrations are noted in the hours directly preceding sunrise (Mazzeo et al. 2005; Kanaya et al. 2007; Warmiński and Rogalski 2007; Kalbarczyk et al. 2016). It has been suggested that the most important night-time reaction takes place between O_3_ and NO. As demonstrated by Nicholson et al. (2001), the drop in O_3_ concentrations in urban air which is characterized by high NO levels is more pronounced in the summer than in the winter. According to our previous research, the NO oxidation rate constant (k_3_) in the city of Olsztyn (north-eastern Poland) ranged from 22.5 to 27.7 ppm^−1^ min^−1^ in July and from 14.1 to 15.5 ppm^−1^ min^−1^ in January (Warmiński and Rogalski 2007). According to Mazzeo et al. (2005), the k_3_ constant in Buenos Aires was 21.5 to 23.0 ppm^−1^ min^−1^ in wintertime. The reported differences are due to various thermal conditions because k_3_ is a function of air temperature as demonstrated by the equation (Seinfeld and Pandis 2016):

2 $$ {k}_3=3.23\cdot {10}^3\exp \left(\frac{-1430}{T}\right)\;\left(\mathrm{pp}{\mathrm{m}}^{-1}\;\mathrm{mi}{\mathrm{n}}^{-1}\right) $$

where *T*—air temperature (K).

Other redox reactions involving O_3_ may be predominant at low NO_x_ concentrations, subject to the locally occurring gaseous pollutants, aerosols, and water vapor. Therefore, we hypothesized that the rate of decrease in tropospheric O_3_ is also influenced by factors other than nitrogen oxide concentrations and temperature. The main motivation for the present study was to assess the sensitivity of night-time ozone concentrations to meteorological parameters at different NO_x_ levels. The results will be useful for modeling tropospheric ozone concentrations. According to Wałaszek et al. (2018), modeling errors are predominantly systematic and result from chemical mechanisms, meteorological fields, and initial and boundary conditions, including background ozone levels. Reliable estimation of initial ozone concentrations is crucial for reducing ozone modeling errors. Ozone decomposition in the lower atmospheric layer is a very important phenomenon which prevents excessive accumulation of this gas in ambient air, in particular on warm summer days.

## Materials and Methods

### Location of the Monitoring Site

**Fig. 1:**
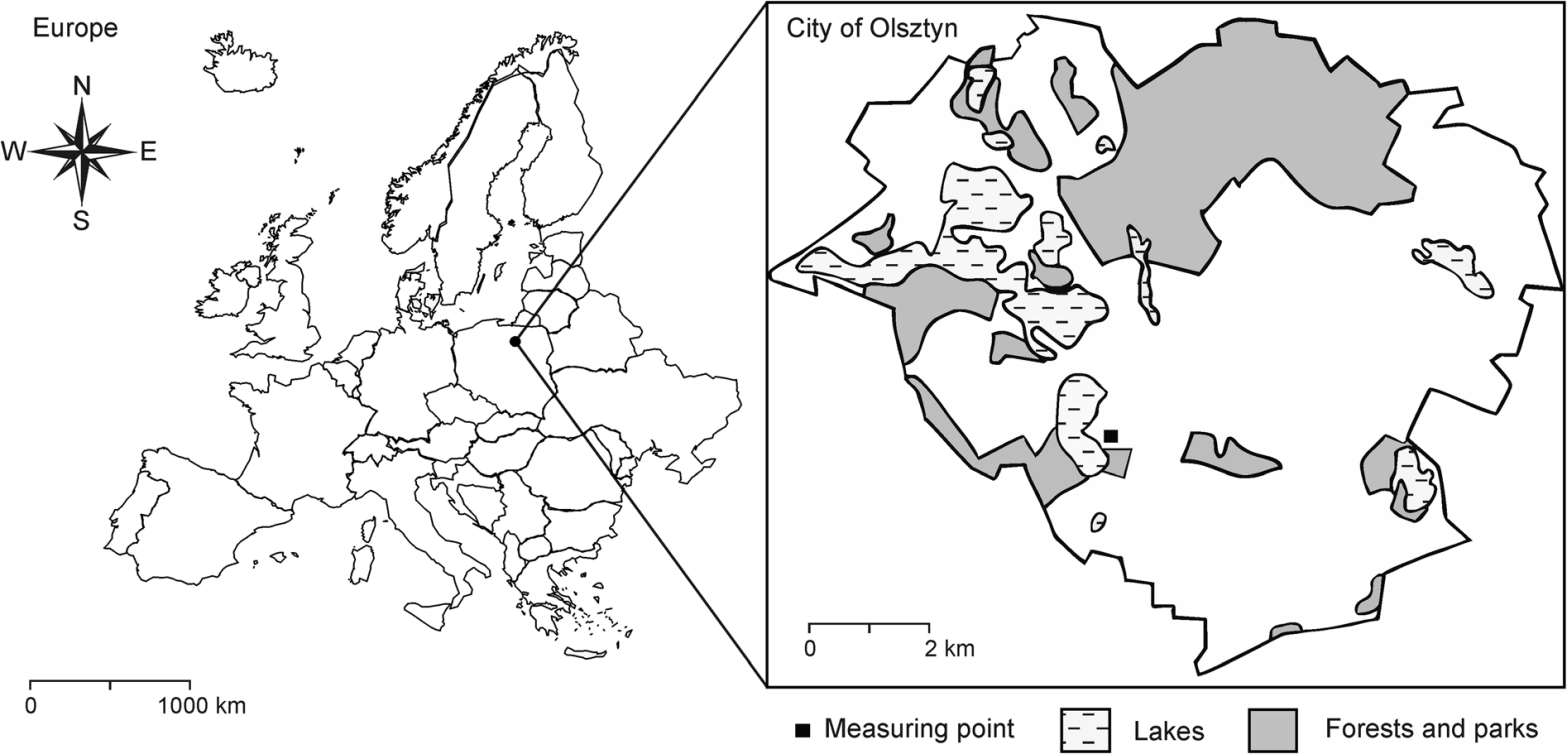
Location of the city of Olsztyn in Europe and map of Olsztyn with an indication of the measuring point

The study was conducted at the air quality monitoring station of the University of Warmia and Mazury in Olsztyn. The station’s location and the mode of collecting representative air samples are consistent with the requirements set forth in the Regulation of the Polish Minister of Environment of September 3, 2012 and Directive 2008/50/EC of the European Parliament and of the Council of May 21, 2008 (EU 2008). The city of Olsztyn is situated in north-eastern Poland, in a region which is popularly known as the Green Lungs of Poland (Fig. 1). The region is characterized by a low degree of industrialization and by exceptional environmental values. The main sources of air pollution in Olsztyn are road transport and household gas emissions. The air quality monitoring station is situated in the south-western part of Olsztyn, on Lake Kortowskie. The monitoring site has the following geographic coordinates: 53° 45′ 34.3″ N, 20° 27′ 09.5″ E. The nearest sources of pollution are Warszawska street which is a part of national road No. 16 (east-bound, at a distance of approx. 500 m), residential estates in Słoneczny Stok composed of single-family houses equipped with coal-fired boilers (1000 m), Dajtki (1400 m), Brzeziny (1600 m), and the city center (2200 m).

The results of daily measurements were compared with data from selected air quality monitoring stations of the Chief Inspectorate for Environmental Protection (GIOŚ) to determine spatiotemporal trends in tropospheric O_3_ and NO_x_ levels. The selected monitoring stations are situated in the Region of Warmia and Mazury (north-eastern Poland), the Warsaw Metropolitan Area (capital of Poland, central Poland), and the Silesian Metropolitan Area (highly industrialized region in southern Poland). The stations in north-eastern Poland are situated in the cities of Olsztyn, Elbląg, Ostróda, Mrągowo, and Gołdap. Data were also obtained from a rural background monitoring station in the Borecka Forest (PL0005R Diabla Gora) which is a part of the European Monitoring and Evaluation Program (EMEP). In the Warsaw Metropolitan Area, O_3_ concentrations were presented as the average value from five monitoring stations in Warsaw, and the results from the Silesian Metropolitan Area were averaged based on the data from the cities of Katowice, Zabrze, Tychy, and Dąbrowa Górnicza. The concentrations of NO_x_ were measured in nine monitoring stations in each region. Detailed information about monitoring stations and measurements is provided by GIOŚ at http://powietrze.gios.gov.pl.

### Measurements and Instrumentation

**Fig. 2:**
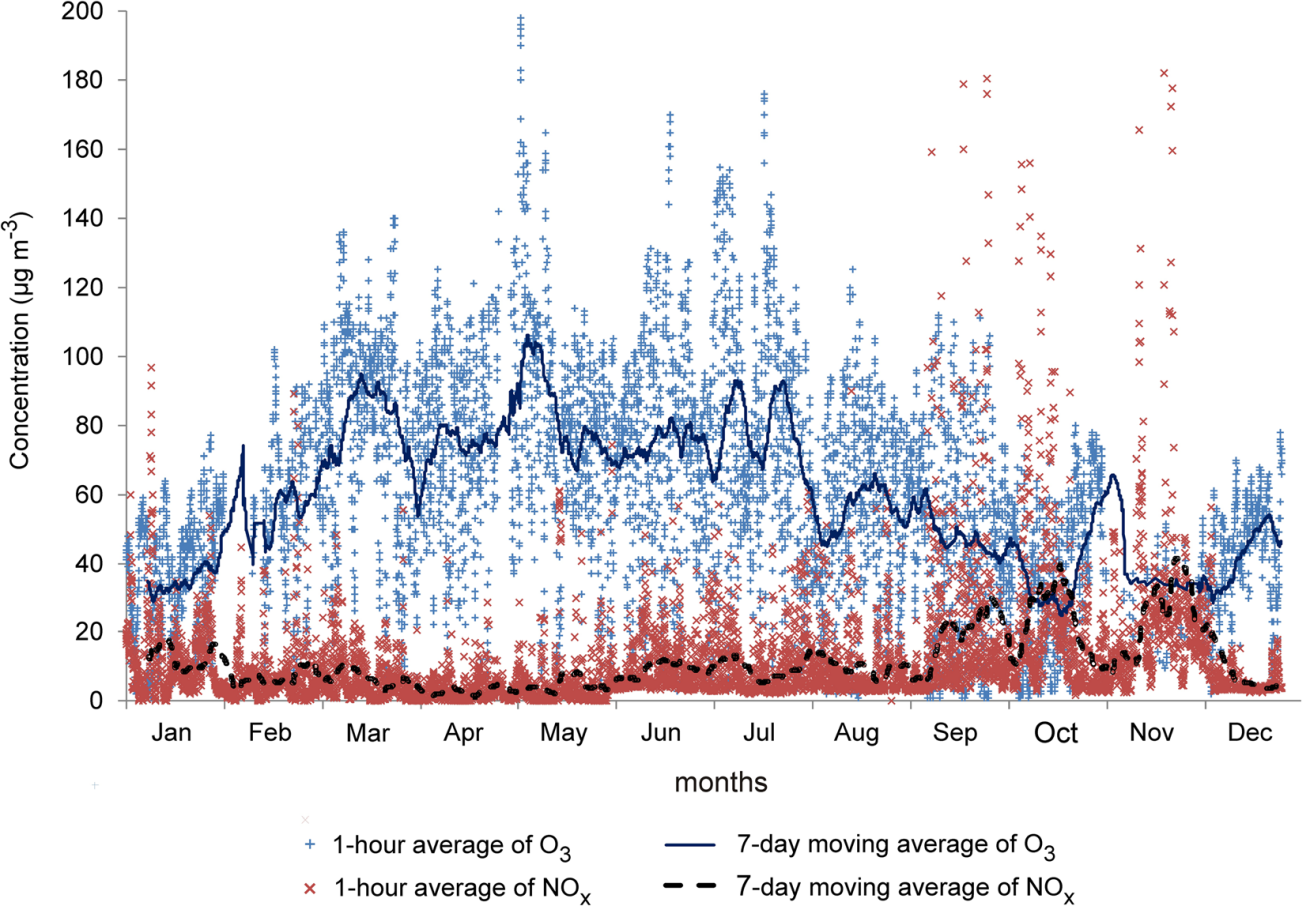
Temporal trends in ground-level O_3_ and NO_x_ concentrations in ambient air in low-polluted urban area of the city of Olsztyn

NO and NO_2_ concentrations were measured continuously by the chemiluminescence method, and O_3_ levels were determined by the UV photometric method (API 200E and API 400E analyzers, Teledyne Advanced Pollution Instrumentation, Inc. USA). The applied methods are consistent with the ISO 7996:1985 and ISO 13964:1998 standards (ISO 1985; ISO 1998), and the analyzers have US EPA certification (USEPA RFNA-1194-099 and USEPA EQOA-0992-087). All analyzers met the requirements of minimum data capture (DC), stipulated by Directive 2008/50/EC (EU 2008). Minimum DC for O_3_ and NO_x_ is 90% in summer and 75% in winter. In our study, conducted in 2006, minimum DC was 98.6 and 76.1% for O_3_ and 98.5 and 91.1% for NO_x_ in summer and winter, respectively. Air temperature and humidity were measured with the DMA580 digital thermo-hygrometer, wind direction and speed measurements were performed using the DNA502 gonio-anemometer and the DNA511 tacho-anemometer, and precipitation was measured with the DQA031 automated rain gauge (LSI s.p.a., Italy). Instantaneous data (5-s) were registered, validated, and averaged to 1 h in a datalogger with the CS5 v. 5.3 system (CSMS, Poland). Temporal trends in ground-level O_3_ and NO_x_ concentrations in ambient air in low-polluted urban area of the city of Olsztyn are presented in Fig. 2. The concentrations of O_3_, NO_2_, and NO and meteorological parameters were measured throughout 2006, but this study accounts only for data collected at night-time, when photochemical processes cease and when only reactions leading to O_3_ decomposition take place.

### Data Analysis

We determined the drop in O_3_ concentrations (–ΔO_3_) in hour C with the use of the equation:

3 $$ -\varDelta {O}_3=-\frac{\varDelta x}{\varDelta t}=-\frac{x_{BC}-{x}_{AB}}{t_C-{t}_B}\;\left(\upmu \mathrm{g}\;{\mathrm{m}}^{-3}\;{\mathrm{h}}^{-1}\right) $$

where *x*_*AB*_, *x*_*BC*_—average O_3_ concentration (μg m^−3^) based on instantaneous measurements between hour A to hour B and hour B to hour C; *t*_*C*_, *t*_*B*_—time (h) at hour C and B from the beginning of the measurement series.

Validated NO, NO_2_, NO_x_, and O_3_ concentrations and meteorological parameters (ambient air temperature, relative and absolute air humidity, wind speed and direction, total atmospheric precipitation) were subjected to a preliminary statistical analysis to verify the hypothesis on the goodness of fit of value distribution to normal distributions. Kolmogorov-Smirnov and chi-square tests invalidated the above hypothesis. Therefore Spearman’s rank correlation test was employed to determine relationships between *–*ΔO_3_ and the investigated factors.

Calculations were performed in several time series: (i) throughout the year (all night-time hours); (ii) hours without atmospheric precipitation; (iii) cool season (period with average weekly temperature of ambient air at night-time < 0 °C; in this study, this criterion was fulfilled over a period of 12 weeks, from 01 Jan. 2006 to 25 Mar. 2006); (iv) warm season (12 warmest weeks in the year, from week 25 to 36, i.e. from 11 Jun. 2006 to 09 Sept. 2006; the average night-time temperature of ambient air in the above period was higher than 13.9 °C); (v) only hours during which specific NO_x_ concentrations were recorded (≤ 1.0, ≤ 2.0, ≤ 3.0, and > 3.0 μg m^−3^).

Coefficients of correlation were calculated, and seasonal and diurnal variations in *–*ΔO_3_ were analyzed. The average weekly value of *–*ΔO_3_ was adopted as the dependent variable and the number of the week in the year as the independent variable. Diurnal variation was analyzed separately for the cool and warm season, and the time of the day (independent variable, *x*) was transformed in line with the following rules: (i) from sunset to midnight, the values of *x* for 4 p.m., 5 p.m., 6 p.m., etc. were set at 4, 5, 6, etc.; (ii) at midnight, the value of × was set at 12; (iii) from midnight to sunrise, the values of *x* for 1 a.m., 2 a.m., 3 a.m., etc. were set at 13, 14, 15, etc. The above transformation was performed to ensure the continuity of *x* value (hours) from sunset to sunrise. The obtained data were verified statistically in STATISTICA 10 software (StatSoft, Inc.).

## Results and Discussion

### Spatiotemporal Trends in Tropospheric Ozone and Nitrogen Oxide Concentrations in Poland

**Fig. 3:**
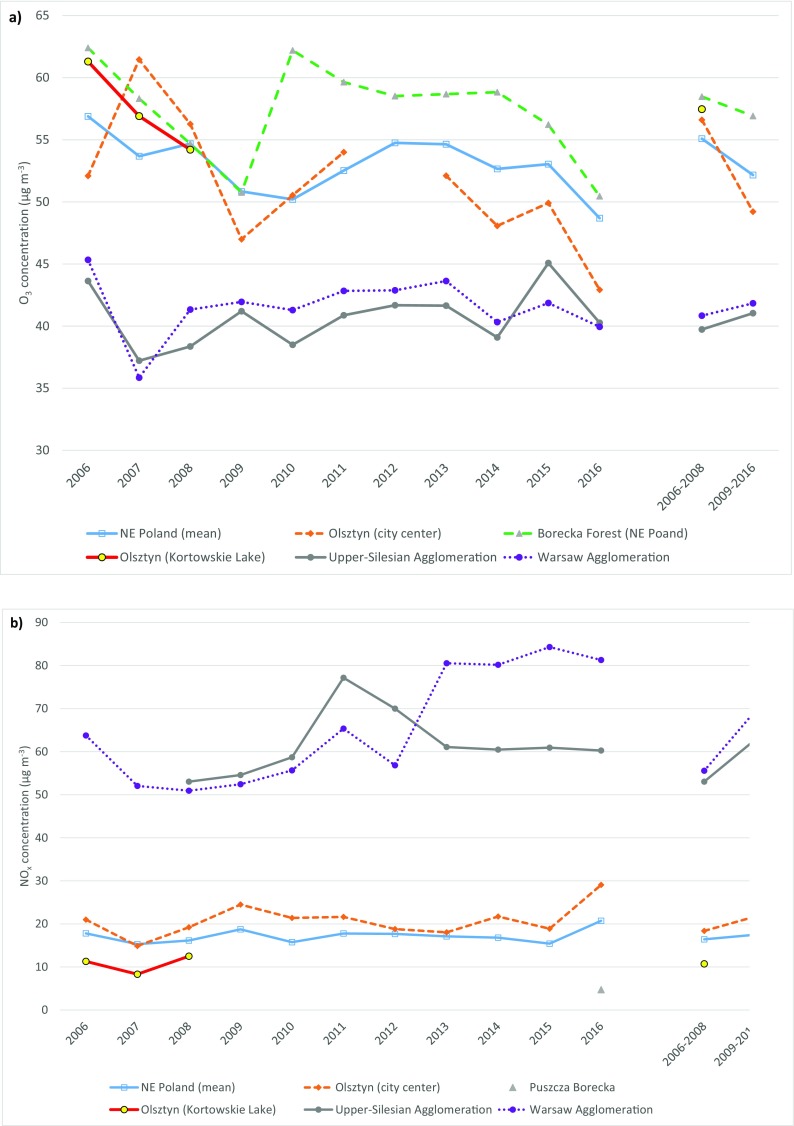
Spatiotemporal trends in ground-level O_3_ and NO_x_ concentrations in selected urban and rural areas in Poland

The average concentration of tropospheric O_3_ in all air quality monitoring stations in the Region of Warmia and Mazury (north-east Poland) was lowest in 2016 and highest in 2006 at 48.7 and 56.9 μg m^−3^, respectively (Fig. 3a). A clear decreasing trend in the above parameter was observed in north-east Poland. In our study, the concentration of tropospheric ozone on Lake Kortowskie in Olsztyn decreased from 61.3 μg m^−3^ in 2006 to 54.2 μg m^−3^ in 2008 and exceeded the regional average by 2.4 μg m^−3^. A similar trend was noted in the rural background monitoring station in the Borecka Forest (EMEP station). In contrast, in the monitoring station in downtown Olsztyn, O_3_ concentration was highest in 2007 at 61.5 μg m^−3^. A comparison of average values for 2006–2008 indicates that O_3_ levels were around 1 μg m^−3^ lower in central Olsztyn than on Lake Kortowskie. The above was related to differences in the concentration of NO_x_ which was around 8 μg m^−3^ higher in central Olsztyn (Fig. 3b). O_3_ and NO_x_ concentrations are measured simultaneously in many locations in Poland and around the world. A negative correlation is observed between ozone and NO_x_ levels due to the reaction between O_3_ and NO (Nicholson et al. 2001; Kalbarczyk et al. 2016). For this reason, ozone levels in industrialized urban areas characterized by higher NO_x_ concentrations, such as Warsaw and the Silesian Metropolitan Area, are approximately 10–11 μg m^−3^ lower than in north-eastern Poland. However, a rising trend in the concentrations of both ozone and nitrogen oxides has been noted in those locations. Kalbarczyk et al. (2016), Karagiannidis et al. (2015), and Mazzeo et al. (2005) reported similar correlations between O_3_ and NO_x_ levels.

### Correlations between –ΔO_3_ vs. Meteorological Factors and Nitrogen Oxide Concentration

The key factors which determined the rate of drop in O_3_ concentrations at night-time were wind speed, total atmospheric precipitation, ambient air temperature, absolute humidity of ambient air, NO_2_, and NO_x_ concentrations (Table 1). Except for wind speed and total atmospheric precipitation, the remaining factors were positively correlated with –ΔO_3_. If data from hours with atmospheric precipitation are omitted in the analysis, the influence of chemical factors on the determination of the dependent variable (–ΔO_3_) decreases, while the impact of meteorological factors increases.

**Table 1:** Relationships between –ΔO_3_ in night-time and independent variables

Independent variables	All night-time hours		Night-time hours without precipitation	
	R_S_	p	R_S_	p
O_3_ concentration	− 0.017	0.327	− 0.013	0.458
NO concentration	0.029	0.087	0.032	0.069
NO_2_ concentration	0.141	< 0.001	0.134	< 0.001
NO_x_ concentration	0.124	< 0.001	0.119	< 0.001
Relative humidity	− 0.023	0.180	− 0.016	0.375
Absolute humidity	0.133	< 0.001	0.142	< 0.001
Temperature	0.149	< 0.001	0.157	< 0.001
Wind direction	− 0.017	0.306	− 0.010	0.564
Wind speed	− 0.313	< 0.001	− 0.323	< 0.001
Precipitation amount	− 0.058	0.001	–	–
Hour of the night	0.099	< 0.001	0.098	< 0.001
Week of the year	− 0.033	0.050	− 0.023	0.191

*R*_*S*_ Spearman’s correlation coefficient, *p* significance level

The obtained results supported our assumptions that temperature significantly contributes to a decrease in O_3_ concentrations. However, no statistically significant correlation was found between the rate of O_3_ concentration drop and input O_3_ and NO concentrations. It could probably be explained by the multifactorial nature of the phenomenon. Moreover, NO concentrations determined at air quality monitoring stations are not input concentrations but the concentrations of NO which has not yet been oxidized to NO_2_. At measuring stations situated at least several hundred meters away from NO_x_ sources, NO is largely oxidized to NO_2_ before it reaches the monitoring station with the air masses (Nicholson et al. 2001; Warmiński and Rogalski 2006). Therefore, NO_x_ concentrations are proportional to initial NO concentration levels. For this reason, no statistically significant dependencies were found between –ΔO_3_ and NO levels, but only between –ΔO_3_ and NO_2_ and NO_x_ concentrations (Table 1). The formation of NO_3_ radicals in dark hours is a well-described reaction which leads to a drop in O_3_ concentrations (Zheng et al. 2017):

R6 $$ {O}_3+N{O}_2\to N{O}_3+{O}_2 $$

NO_3_ radicals are determined practically only at night-time because they rapidly photolyze to NO_2_ and O (Atkinson and Arey 2003, Stutz et al. 2010, Wang et al. 2013, Akimoto 2016).

The highest correlation was found between wind speed and –ΔO_3_, and the determined dependency was negative. Strong winds cause intense mixing of masses of polluted and clean air; therefore, the higher the wind speed, the greater the drop in NO_x_ levels (Table 2). The negative correlation between NO_x_ concentrations and wind speed has been documented in the works of, among others, Delaney and Dowding (1998), Tang et al. (2011), Karagiannidis et al. (2015), and Valotto and Varin (2016). For this reason, we determined lower NO_x_ concentrations and –ΔO_3_ values under strong wind conditions (Tables 1 and 2). A negative correlation between O_3_ concentrations and wind speed was also reported by Banta et al. (2011) and Kalbarczyk et al. (2016).

**Table 2:** Results of Spearman’s correlation analysis investigating the relationship between chemical parameters of ambient air and meteorological factors in night-time in the cool and warm season

Variables	AH	T	WD	WS	P
Cool season					
O_3_ concentration	0.284*	0.397*	0.093*	0.351*	− 0.104*
NO concentration	− 0.109*	− 0.145*	− 0.209*	− 0.126*	0.060
NO_2_ concentration	− 0.408*	− 0.388*	− 0.169*	− 0.482*	− 0.036
NO_x_ concentration	− 0.412*	− 0.394*	− 0.177*	− 0.489*	− 0.032
Warm season					
O_3_ concentration	0.198*	0.458*	0.241*	0.468*	0.058
NO concentration	− 0.178*	− 0.286*	− 0.091*	− 0.166*	0.015
NO_2_ concentration	0.013	0.070	− 0.271*	− 0.731*	− 0.161*
NO_x_ concentration	− 0.017	0.026	− 0.278*	− 0.734*	− 0.151*

Cool season—a period with average weekly temperature of ambient air at night-time of < 0 °C; warm season—12 warmest weeks in the year

*AH* absolute humidity, *T* temperature, *WD* wind direction, *WS* wind speed, *P* total precipitation

*Spearman’s correlation coefficients marked with an asterisk are statistically significant at *p* = 0.01

A positive correlation between –ΔO_3_ and absolute humidity could be due to the reaction between O_3_ and water in the gaseous state. However, this mechanism of O_3_ breakdown at night-time is doubtful due to a very low value of the correlation coefficient at 0.133 (Table 1). Other mechanisms could be involved in the decomposition of ground-level ozone, but the reaction with NO (R3) appears to be predominant.

Ozone and particles adsorbed on aerosol surface also react at a faster rate in high humidity air. Chughtai et al. (2003) proposed the following relationship between the drop in O_3_ concentrations (−d[O_3_]/dt) caused by its reaction with soot aerosol and absolute humidity of air:

4 $$ \frac{-d\left[{O}_3\right]}{dt}={k}^{"}{\left[{O}_3\right]}^2{p}^{0.2} $$

where

[O_3_]: ozone concentrations (ppm)
*p*: absolute humidity (Pa)
*k*″: reaction constant determined by temperature and the type of aerosol surface

Equation 4 indicates that the higher the air humidity, the higher the rate of O_3_ reaction with soot components. Sakamoto et al. (2004) also noted that the presence of water vapor in air speeds up the oxidation of SO_2_ through O_3_ on the surface of yellow sand particles.

Buckley and Birks (1995) also pointed to the possibility of visible-light photolysis of O_3_∙H_2_O cluster molecules (Van der Waals complexes) as well as reaction without the involvement of solar radiation:

R7 $$ {O}_3\cdot {H}_2O\overset{h\upsilon (vis)}{\to }2 OH+{O}_2 $$

R8 $$ {O}_3\cdot {H}_2O\overset{h\upsilon (vis)/ dark}{\to }{H}_2{O}_2+{O}_2 $$

Reaction R7 is energetically possible for light with a wavelength under 665 nm, while reaction R8 is exothermic and it takes place even without light absorption, i.e., at night. Those reactions could also be responsible for higher –ΔO_3_ values observed at night-time when air humidity is high.

Air pollutants, in particular the highly reactive O_3_, can also undergo dry deposition on the surface of soil, plants, buildings, etc. During the day, in vegetated areas, both stomatal fluxes and non-stomatal fluxes play an important role in the dry deposition of O_3_. At night, the dry deposition of O_3_ is dominated by non-stomatal fluxes (Fares et al. 2010; El-Madany et al. 2017). The total dry deposition of O_3_ is several-fold or 10- to 20-fold lower at night than during the day (Padro 1996; El-Madany et al. 2017). According to the literature, stomatal flux is proportional to air humidity (Zapletal et al. 2012; Fares et al. 2013). The night-time concentrations of O_3_ decrease steadily due to the absence of sunlight and redox reactions.

Total atmospheric precipitation was negatively correlated with –ΔO_3_ (Table 1). Although the Spearman’s rank correlation coefficient was statistically significant (*p* < 0.01), the precipitation factor in the analysis of annual data did not produce satisfactory results (R_S_ = − 0.058). We propose a hypothesis that this is due to the types of precipitation at various times of the year. The analysis of annual data accounts for total precipitation which is not broken into different types of precipitation, i.e., rain, snow, or hail. For this reason, we performed an additional analysis of correlation covering two seasons: (i) cool (marked by ambient air temperature below 0 °C; coldest 12 weeks in 2006) and (ii) warm (12 warmest weeks in 2006).

**Fig. 4:**
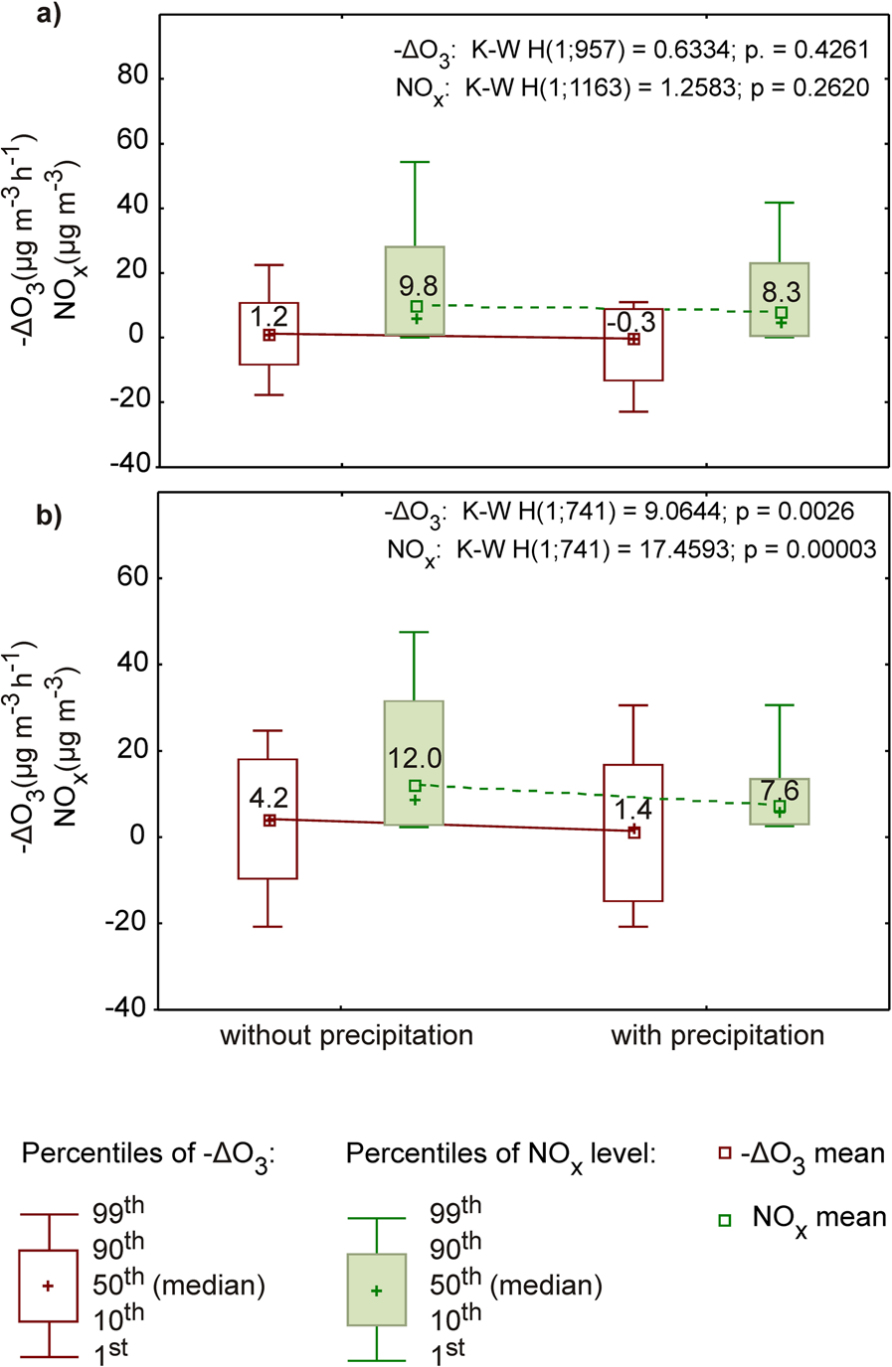
The effect of atmospheric precipitation on –ΔO_3_ and NO_x_ concentrations at night-time hours in the cool (**a**) and warm (**b**) season

The correlation between –ΔO_3_ and total atmospheric precipitation was higher in the warm period (R_S_ = − 0.113, *p* < 0.001) in comparison with data covering the entire year (Table 3). A negative correlation rank coefficient may seem puzzling in this context. It indicates that O_3_ concentrations decrease at a slower rate as precipitation intensity grows. An analysis of data showed that at times of precipitation over the warm season, –ΔO_3_ reaches 1.43 μg m^−3^ h^−1^ on average, while it is threefold higher in hours without precipitation (Fig. 4). This is probably due to lower NO_x_ concentrations at times of precipitation and, as indicated earlier, NO significantly contributes to a reduction in tropospheric O_3_ levels. It should be noted that the value of R_s_ was very low, although statistically significant. A stronger correlation between –ΔO_3_ and NO_2_ (NO_x_) concentrations and wind speed was determined in the warm season than in the cool season (Table 3). However, statistically significant correlations between –ΔO_3_ and air temperature and between –ΔO_3_ and air humidity were not found in either of the analyzed periods.

**Table 3:** Relationships between –ΔO_3_ in night-time and independent variables in the cool and warm season

Independent variables	Cool season		Warm season	
	R_S_	p	R_S_	p
O_3_ concentration	0.001	0.973	− 0.061	0.100
NO concentration	0.038	0.237	0.021	0.567
NO_2_ concentration	0.156	< 0.001	0.273	< 0.001
NO_x_ concentration	0.154	< 0.001	0.262	< 0.001
Relative humidity	− 0.047	0.142	− 0.046	0.213
Absolute humidity	− 0.064	0.045	0.069	0.060
Temperature	− 0.044	0.170	0.079	0.032
Wind direction	0.065	0.042	− 0.068	0.064
Wind speed	− 0.212	<0.001	− 0.388	< 0.001
Precipitation amount	− 0.025	0.447	− 0.113	0.002
Hour of the day	0.086	0.007	0.129	< 0.001
Week of the year	0.160	< 0.001	− 0.194	< 0.001

*R*_*S*_ Spearman’s correlation coefficient, *p* significance level

### Influence of NO_x_ Concentrations on the Results of Correlation Analysis

Nitrogen oxide concentrations proved to be one of the most important factors determining the rate of the drop in O_3_ concentrations at night-time. In view of the above, an additional Spearman’s rank correlation test was performed for night-time data, and the obtained average NO_x_ concentrations were in the range of ≤ 1.0, ≤ 2.0, ≤ 3.0, and > 3.0 μg m^−3^. An analysis of correlation test results in Table 4 indicates that selected meteorological factors have a growing impact on –ΔO_3_ values as NO_x_ concentrations decrease from > 3.0 to ≤ 1.0 μg m^−3^. It applies primarily to temperature and absolute humidity and, to a lesser extent, to wind speed. A reverse dependency was reported for NO_2_ and NO_x_ concentrations.

**Table 4:** Effect of NO_x_ concentrations on the results of Spearman’s correlation analysis between –ΔO_3_ (dependent variable) and the investigated factors (independent variables)

Independent variables	Spearman’s correlation coefficient (R_S_) for night-time hours with NO_x_ concentration within the following ranges			
	≤ 1.0 (μg m^−3^)	≤ 2.0 (μg m^−3^)	≤ 3.0 (μg m^−3^)	> 3.0 (μg m^−3^)^a^
O_3_ concentration	−0.019	− 0.098	− 0.014	0.004
NO concentration	0.120	− 0.009	− 0.052	0.011
NO_2_ concentration	0.019	0.093	0.115*	0.130*
NO_x_ concentration	0.057	0.078	0.042	0.123*
NO/NO_x_ ratio	0.154	0.019	− 0.038	− 0.078*
Relative humidity	− 0.007	0.015	− 0.020	− 0.031
Absolute humidity	0.243*	0.214*	0.178*	0.128*
Temperature	0.271*	0.214*	0.184*	0.144*
Wind direction	0.098	0.031	0.038	− 0.024
Wind speed	− 0.376*	− 0.349*	− 0.321*	− 0.306*
Precipitation amount	− 0.085	− 0.030	− 0.082	− 0.057
Hour of the day	0.103	0.097	0.067	0.098*
Week of the year	0.331*	0.205*	0.173*	− 0.091*
Number of samples	172	314	543	2894

*Spearman’s correlation coefficients marked with an asterisk are statistically significant at *p* = 0.01

^a^Threshold concentration level above which significant correlations between –ΔO_3_ and NO_x_ concentrations were determined

### Seasonal and Diurnal Variations of –ΔO_3_

**Fig. 5:**
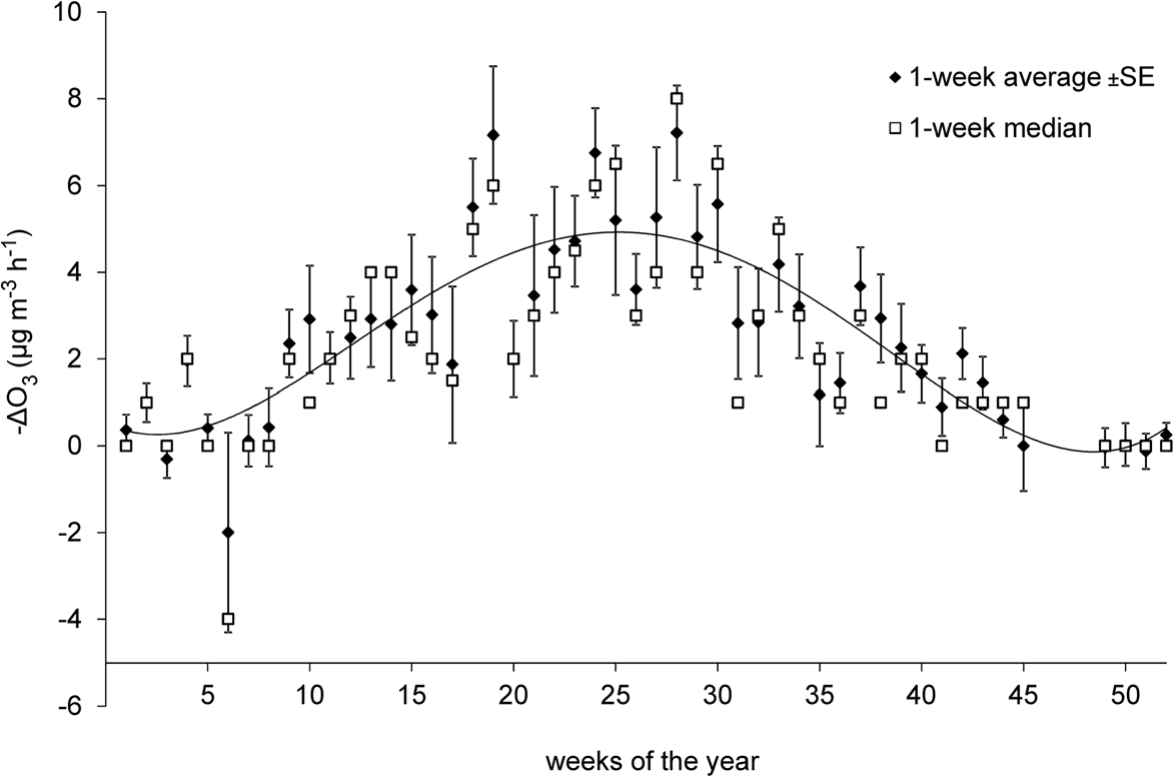
Seasonal variation of –ΔO_3_ at night-time hours in 2006 (1-week average and 1-week median)

Tables 1, 3, and 4 present Spearman’s rank correlation coefficients (R_S_) and significance levels (p) determined not only between –ΔO_3_ and chemical and meteorological factors, but also between –ΔO_3_ and the hour of the day and the week of the year. In most cases, those coefficients were statistically significant. Seasonal variations in –ΔO_3_ (weekly data) are presented in Fig. 5. Maximum –ΔO_3_ values were noted in the summer, whereas minimum ΔO_3_ values (near zero) were observed at the beginning and end of the year. The noted difference is due mostly to seasonal variations in air temperature.

**Fig. 6:**
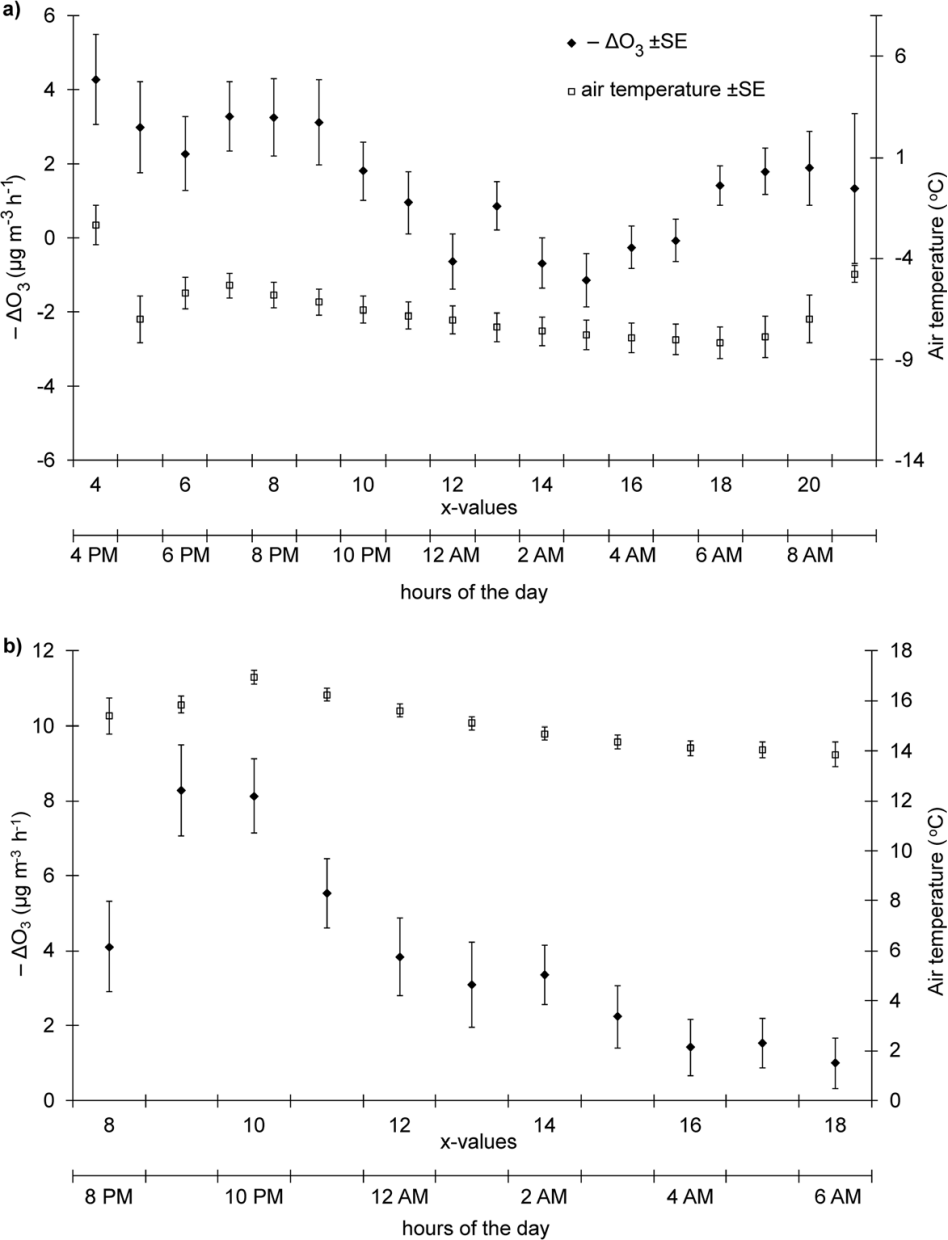
Diurnal variation of –ΔO_3_ and air temperature at night-time in the cool (**a**) and warm season (**b**). *x*-value is the transformed night-time hour (*x* = 0 for 12 PM and *x* = 12 for 12 AM)

In the cool period with temperatures of < 0 °C, diurnal changes in –ΔO_3_ values ranged from around − 1.0 to 4.0 μg m^−3^ h^−1^ (Fig. 6a). Higher fluctuations in –ΔO_3_ values were observed in the warm period, from around 1.0 to more than 8.0 μg m^−3^ h^−1^ (Fig. 6b). The highest –ΔO_3_ values were recorded between 4 p.m. and 9 p.m. in the cool season and between 9 p.m. and 10 p.m. in the warm season. The highest drop in O_3_ concentrations is observed in the hours directly after sunset, when air temperature is still relatively high but photochemical processes have already ceased. In the following hours, –ΔO_3_ values gradually decrease. OH and HO_2_ radical concentrations also begin to decrease as of sunset (Arias and Hastie 1996; Cantrell et al. 1996; Commane et al. 2010).

## Conclusions

Ozone decomposition reactions in the troposphere are an important stage of the O_3_ cycle. The higher the rate of those reactions, the lower the risk of photochemical smog. The analysis of changes in concentration levels of O_3_ and other air parameters in the lower parts of the troposphere at night-time supported the preliminary identification of factors which significantly contribute to the rate of those reactions in low-polluted urban area. We conclude that the rate of decomposition of tropospheric ozone (–ΔO_3_) was affected mainly by the presence of NO_x_ and wind speed. It should be noted, however, that NO_x_ concentrations were largely determined by wind speed. High wind speed causes intense mixing of air masses, leading to a drop in the concentration levels of pollutants in urban areas, including NO_x_. Therefore, wind speed indirectly influenced –ΔO_3_. Other significant determinants included air temperature and absolute humidity which were positively correlated with –ΔO_3_. Maximum correlation coefficients for these parameters were observed at low NO_x_ concentrations in the air (≤ 1.0 μg m^−3^), which reached 0.271 and 0.243, respectively. Both values of R_S_ were similar to the value of R_S_ for NO_x_. It should be noted that correlation coefficients were low, but statistically significant. The above could suggest that ozone also reacts with air components other than NO_x_. The observation that the decrease in ozone concentrations was not correlated with relative humidity, but was correlated with absolute humidity, in particular when NO_x_ levels were very low, is a crucial finding. The effect of absolute humidity on –ΔO_3_ decreased with a rise in NO_x_ levels. Precipitation in the cool season with temperatures of < 0 °C did not contribute to a drop in O_3_ concentrations at night-time. In the warm season, rainfalls slowed down the rate at which O_3_ concentrations decreased, compared with dry spells. This could result from effective removal of NO_x_, in particular the highly water-soluble NO_2_, by rain. O_3_ is sparingly water-soluble, and its wet deposition takes longer relative to NO_2_. Seasonal and daily fluctuations in temperature were responsible for –ΔO_3_ variations. The highest –ΔO_3_ values were reported in the warmest period of the year and shortly after sunset in the daily cycle. The presence of high concentration levels of OH and HO_2_ radicals directly after sunset could also be a cause of the observed variations in –ΔO_3_ values at that time.

To conclude, it can be postulated that some factors which significantly affect O_3_ formation during day-time (nitrogen oxides, high air temperature, low wind speed) also contribute to the rate of O_3_ decomposition at night-time. For this reason, daily O_3_ concentration amplitudes are much higher in the summer than at other times of the year. We are also of the opinion that low air humidity during the summer continental high slows down the drop in O_3_ concentrations at night-time. Day-time weather in the same period is marked by high temperatures and insolation, which supports O_3_ formation in photochemical processes. Due to the above, O_3_ concentrations may exceed target value for protection of human health after only several hot summer days and nights with low air humidity.

Our results can be useful for improving tropospheric ozone models. Such models account for ozone forming parameters as well as initial ozone levels (Wałaszek et al. 2018). As demonstrated by the study, selected meteorological parameters and NO_x_ levels have a small, but statistically significant effect on initial ozone levels.

## References

[CR1] Akimoto H (2016). Atmospheric reaction chemistry.

[CR2] Arias M, Hastie D (1996). Radical chemistry at the SONTOS site in rural Ontario. Atmospheric Environment.

[CR3] Atkinson R, Arey J (2003). Gas-phase tropospheric chemistry of biogenic volatile organic compounds. A review. Atmospheric Environment.

[CR4] Banta R, Senff C, Alvarez R, Langford A, Parrish D, Trainer M (2011). Dependence of daily peak O_3_ concentrations near Houston, Texas on environmental factors: wind speed, temperature, and boundary-layer depth. Atmospheric Environment.

[CR5] Buckley P, Birks J (1995). Evaluation of visible-light photolysis of ozone-water cluster molecules as a source of atmospheric hydroxyl radical and hydrogen peroxide. Atmospheric Environment.

[CR6] Cantrell C, Shetter R, Calvert J (1996). Peroxy radical chemistry during FIELDVOC 1993 in Brittany, France. Atmospheric Environment.

[CR7] Chughtai A, Kim J, Smith D (2003). The effect of temperature and humidity on the reaction of ozone with combustion soot: implications for reactivity near the tropopause. Journal of Atmospheric Chemistry.

[CR8] Commane R, Floquet C, Ingham T, Stone D, Evans M, Heard D (2010). Observations of OH and HO_2_ radicals over West Africa. Atmospheric Chemistry and Physics.

[CR9] Crutzen P, Lawrence M, Pöschl U (1999). On the background photochemistry of tropospheric ozone. Tellus B: Chemical and Physical Meteorology.

[CR10] de Wit, H., Hettelingh, J.-P., & Harmens, H. (2015). *Trends in ecosystem and health responses to long-range transported atmospheric pollutants. ICP waters report 125/2015.* Norway: Norwegian Institute for Water Research. Oslo.

[CR11] Delaney C, Dowding P (1998). The relationship between extreme nitrogen oxide (NOx) concentrations in Dublin’s atmosphere and meteorological conditions. Environmental Monitoring and Assessment.

[CR12] EEA. (2017). *Air quality in Europe—2017 report.* EEA report, no 13/2017*.* Luxembourg: European Environment Agency, Publications Office of the European Union.

[CR13] El-Madany T, Niklasch K, Klemm O (2017). Stomatal and non-stomatal turbulent deposition flux of ozone to a managed peatland. Atmosphere.

[CR14] EU. (2008). Directive 2008/50/EC of the European Parliament and of the Council of 21 May 2008 on ambient air quality and cleaner air for Europe. *Official Journal of the European Union,* L 152/1.

[CR15] Fares S, McKay M, Holzinger R, Goldstein A (2010). Ozone fluxes in a Pinus ponderosa ecosystem are dominated by non-stomatal processes: evidence from long-term continuous measurements. Agricultural and Forest Meteorology.

[CR16] Fares S, Matteucci G, Scarascia Mugnozza G, Morani A, Calfapietra C, Salvatori E (2013). Testing of models of stomatal ozone fluxes with field measurements in a mixed Mediterranean forest. Atmospheric Environment.

[CR17] Harmens H, Mills G, Hayes F, Sharps K, Frontasyeva M (2016). Air pollution and vegetation. ICP Vegetation Annual Report 2015/2016.

[CR18] Holland M, Kinghorn S, Emberson L, Cinderby S, Ashmore M, Mills G (2006). Development of a framework for probabilistic assessment of the economic losses caused by ozone damage to crops in Europe. ICP Vegetation Report.

[CR19] Hosoi S, Yoshikado H, Gaidajis G, Sakamoto K (2011). Study of the relationship between elevated concentrations of photochemical oxidants and prevailing meteorological conditions in the North Kanto area, Japan. Water, Air, & Soil Pollution.

[CR20] IPCC. (2007). Climate change 2007: the physical science basis*. Contribution of Working Group I to the Fourth Assessment Report of the IPCC.* Intergovernmental Panel on Climate Change. Cambridge and New York.

[CR21] ISO. (1985). Ambient air—determination of the mass concentration of nitrogen oxides—chemiluminescence method*.* ISO 7996:1985. International Organisation for Standardisation. Geneva.

[CR22] ISO. (1998). Air quality—determination of ozone in ambient air—ultraviolet photometric method*.* ISO 13964:1998. International Organisation for Standardisation. Geneva.

[CR23] Kalbarczyk R, Kalbarczyk E (2017). Variability and temporal structure of concentrations of carbon monoxide in Poznań (Central-Western Poland). Journal of Elementology.

[CR24] Kalbarczyk R, Sobolewski R, Kalbarczyk E (2016). Biometeorological determinants of the tropospheric ozone concentration in the suburban conditions of Wroclaw, Poland. Journal of Elementology.

[CR25] Kanaya Y, Tanimoto H, Matsumoto J, Furutani H, Hashimoto S, Komazaki Y (2007). Diurnal variations in H_2_O_2_, O_3_, PAN, HNO_3_ and aldehyde concentrations and NO/NO_2_ ratios at Rishiri Island, Japan: potential influence from iodine chemistry. The Science of the Total Environment.

[CR26] Karagiannidis A, Poupkou A, Giannaros T, Giannaros C, Melas D, Argiriou A (2015). The air quality of a Mediterranean urban environment area and its relation to major meteorological parameters. Water, Air, & Soil Pollution.

[CR27] Leighton P (1961). Photochemistry of air pollution.

[CR28] Manahan S (2007). Environmental science and technology. A sustainable approach to green science and technology.

[CR29] Mazzeo N, Venegas L, Choren H (2005). Analysis of NO, NO_2_, O_3_ and NO_x_ concentrations measured at a green area of Buenos Aires City during wintertime. Atmospheric Environment.

[CR30] Nicholson J, Weston K, Fowler D (2001). Modelling horizontal and vertical concentration profiles of ozone and oxides of nitrogen within high-latitude urban areas. Atmospheric Environment.

[CR31] Padro J (1996). Summary of ozone dry deposition velocity measurements and model estimates over vineyard, cotton, grass and deciduous forest in summer. Atmospheric Environment.

[CR32] Sakamoto K, Takada H, Sekiguchi K (2004). Influence of ozone, relative humidity, and flow rate on the deposition and oxidation of sulfur dioxide on yellow sand. Atmospheric Environment.

[CR33] Seinfeld J, Pandis S (2016). Atmospheric chemistry and physics: From air pollution to climate change.

[CR34] Sharma S, Sharma P, Khare M (2017). Photo-chemical transport modelling of tropospheric ozone: a review. Atmospheric Environment.

[CR35] Stutz J, Wong K, Lawrence L, Ziemba L, Flynn J, Rappenglück B (2010). Nocturnal NO_3_ radical chemistry in Houston, TX. Atmospheric Environment.

[CR36] Tang L, Rayner D, Haeger-Eugensson M (2011). Have meteorological conditions reduced NO_2_ concentrations from local emission sources in Gothenburg?. Water, Air, & Soil Pollution.

[CR37] Valotto G, Varin C (2016). Characterization of hourly NOx atmospheric concentrations near the Venice international airport with additive semi-parametric statistical models. Atmospheric Research.

[CR38] Wałaszek K, Kryza M, Werner M (2018). The role of precursor emissions on ground level ozone concentration during summer season in Poland. Journal of Atmospheric Chemistry.

[CR39] Wang S, Shi C, Zhou B, Zhao H, Wang Z, Yang S (2013). Observation of NO_3_ radicals over Shanghai, China. Atmospheric Environment.

[CR40] Warmiński K, Bęś A (2009). Diurnal and seasonal variations in the NO_2_ photolysis rate constant, NO titration rate constant and the NO_2_/NO ratio in ambient air in the City of Olsztyn. Ecological Chemistry and Engineering A.

[CR41] Warmiński K, Rogalski L (2006). Wind direction as a determinant of nitric oxide and nitrogen dioxide imission. Zeszyty Problemowe Postepow Nauk Rolniczych.

[CR42] Warmiński K, Rogalski L (2007). Analysis of photochemical reactions of ozone and its precursors in the troposphere in the summer and winter periods. Polish Journal of Environmental Studies.

[CR43] Zapletal M, Pretel J, Chroust P, Cudlín P, Edwards-Jonášová M, Urban (2012). The influence of climate change on stomatal ozone flux to a mountain Norway spruce forest. Environmental Pollution.

[CR44] Zheng S, Singh R, Wu Y, Wu C (2017). A comparison of trace gases and particulate matter over Beijing (China) and Delhi (India). Water, Air, & Soil Pollution.

